# Visual Feedback Is Not Necessary for the Learning of Novel Dynamics

**DOI:** 10.1371/journal.pone.0001336

**Published:** 2007-12-19

**Authors:** David W. Franklin, Udell So, Etienne Burdet, Mitsuo Kawato

**Affiliations:** 1 National Institute of Information and Communications Technology, Keihanna Science City, Kyoto, Japan; 2 ATR Computational Neuroscience Laboratories, Keihanna Science City, Kyoto, Japan; 3 Department of Engineering, University of Cambridge, Cambridge, United Kingdom; 4 Department of Bioengineering, Imperial College London, London, United Kingdom; Lund University, Sweden

## Abstract

**Background:**

When learning to perform a novel sensorimotor task, humans integrate multi-modal sensory feedback such as vision and proprioception in order to make the appropriate adjustments to successfully complete the task. Sensory feedback is used both during movement to control and correct the current movement, and to update the feed-forward motor command for subsequent movements. Previous work has shown that adaptation to stable dynamics is possible without visual feedback. However, it is not clear to what degree visual information during movement contributes to this learning or whether it is essential to the development of an internal model or impedance controller.

**Methodology/Principle Findings:**

We examined the effects of the removal of visual feedback during movement on the learning of both stable and unstable dynamics in comparison with the case when both vision and proprioception are available. Subjects were able to learn to make smooth movements in both types of novel dynamics after learning with or without visual feedback. By examining the endpoint stiffness and force after learning it could be shown that subjects adapted to both types of dynamics in the same way whether they were provided with visual feedback of their trajectory or not. The main effects of visual feedback were to increase the success rate of movements, slightly straighten the path, and significantly reduce variability near the end of the movement.

**Conclusions/Significance:**

These findings suggest that visual feedback of the hand during movement is not necessary for the adaptation to either stable or unstable novel dynamics. Instead vision appears to be used to fine-tune corrections of hand trajectory at the end of reaching movements.

## Introduction

In order to perform motor tasks, the central nervous system integrates multiple modes of sensory information [1]–[3], particularly vision and proprioception. The weighting of this integration may vary depending on the task [4], [5]. Similarly, vision and proprioception contribute differently to different components of the motor command [6]. The performance and learning of novel reaching tasks requires people to integrate multi-modal sensory information about the new environment in order to learn to apply the appropriate forces necessary to compensate for the novel dynamics. When learning a novel task, an array of sensory modalities and information could be used. Visual information provides contextual cues, trajectory feedback, and information about the appearance of objects in the environment. In addition to visual information, muscle spindles, Golgi tendon organs, joint and tactile sensors provide proprioceptive and tactile information about the person's body and any object which is now coupled to the body. For example, if a person picks up a screwdriver for the first time, the entire system, composed of the person, their limbs and the screwdriver, now has altered dynamics due to the added mass and inertia of the tool. The tool also drastically modifies the interaction with the environment, which can become unstable. These effects change the required muscle activation patterns throughout the entire system in order to move the tool appropriately. The person therefore must learn how to compensate for these new dynamics in order to be able to effectively use the tool. By practicing and using the combined visual, proprioceptive and contextual feedback information available to him, the person can gradually learn to adjust the necessary forces or joint torques produced by his arms in order to perform the movement correctly. The question arises as to whether all of these modes of sensory feedback are necessary or important for learning an internal model of these novel dynamics. What sensory signals actually drive this adaptation?

It has been shown that people are able to learn the novel dynamics of both stable environments as well as unstable or unpredictable environments [7]–[10]. However, the types of compensation required in stable and unstable interactions are different, and learning these two kinds of interactions may involve distinct processes [11], [12]. While succeeding in stable dynamics requires the production of counteracting interaction forces through the feed-forward motor command, the unpredictability brought by unstable interactions requires modifying the limb impedance. Evidence suggests that in stable dynamics an internal model of the environmental forces is acquired [7], [13] while in unstable or unpredictable dynamics impedance control may be used to selectively modify endpoint impedance through the co-activation of specific muscle pairs [10], [14]–[17].

While both visual and somatosensory feedback are likely to play a critical role in the learning of novel dynamics, several previous studies on motor learning have shown that adaptation to stable dynamics is possible even when subjects are provided only with delayed visual feedback of the trajectory [18], deprived of online visual feedback of their hand position during movements [19], [20] or are congenitally blind [21]. What is the role of visual feedback during movement when learning and performing in unstable or unpredictable environments? As learning unstable dynamics is significantly more difficult than learning stable dynamics [12], the first question arising is whether subjects are able to compensate for unstable dynamics without vision of the limb position during movement. Is visual feedback necessary for learning, and does on-line stabilization of movement after learning require visual feedback? If such motor learning depends primarily on somatosensory feedback then it should also be possible to compensate for unstable dynamics without visual feedback. To address these questions we observed the learning of horizontal planar reaching movements in an unstable divergent force field (DF) with and without visual feedback of hand position during movement. Learning of a stable velocity-dependent curl force field (CF) with and without vision was also examined for comparison. Endpoint stiffness after adaptation was measured to investigate the potential contribution of online visual feedback to any differences in limb impedance.

## Materials and Methods

### Experimental Methods

#### Subjects

Eight (6 male and 2 female) neurologically normal subjects participated in the study (mean age: 25±4 years). All subjects were right-handed according to the Edinburgh handedness inventory [22]. All subjects had previously participated in similar motor control studies. Subjects gave verbal informed consent and the experiments were approved by the institutional ethics committee (ATR Ethics Committee).

#### Apparatus

**Figure 1:**
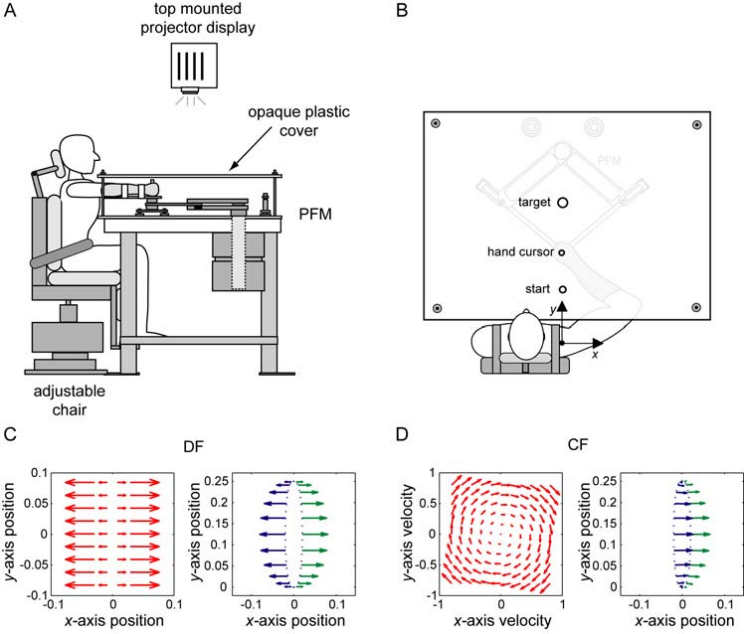
The experimental setup. (A) A side view. The subject is attached to the robotic manipulandum with a custom fitted thermoplastic cuff. An opaque plastic tabletop covers the manipulandum, arm and hand of the subject preventing any visual information of their location. Visual feedback of the targets and hand position are presented using a top mounted projector onto the plastic sheet. (B) A top view of the setup showing the targets displayed on top of the plastic cover. On this diagram the PFM and arm are shown visible through the material simply for illustration purposes. (C) The unstable divergent force field (DF). On the left, the force field is shown as a function of the hand position. On the right side, the forces applied to hand are shown for two slightly different paths to either side of the straight line joining the start and end targets. Small differences in the trajectory produce large differences in the forces applied to the hand. (D) The stable curl force field (CF). On the left, the force field is shown as a function of the hand velocity. On the right side, the forces applied to hand are shown for two slightly different paths to either side of the straight line joining the start and end targets. Small differences in the trajectory produce almost no difference in the forces applied to the hand.

Subjects were seated with their shoulders restrained against the back of a chair by a shoulder harness. A custom-molded rigid thermoplastic cuff was securely fastened around the subjects' right wrist and forearm, immobilizing the wrist joint. Only the shoulder and elbow joints remained free to move in the horizontal plane. The subjects' forearm was secured to a support beam in the horizontal plane and the cuff and beam were coupled to the handle of the parallel-link direct drive air-magnet floating manipulandum (PFM) used to generate the environmental dynamics (Figure 1). Movement was thus restricted to a single degree of freedom in each joint in the horizontal plane. The PFM was powered by two DC direct-drive motors controlled at 2 kHz and the subjects' hand position was measured using optical joint position sensors (409,600 pulse/rev). The force applied by subjects at the handle of the PFM was measured using a six-axis force-torque sensor (Nitta Corp. No. 328) with a resolution of 0.06 N. Position and force data were sampled at 500 Hz. The handle of the PFM (subjects' hand position) was supported by a frictionless air-magnet floating mechanism. The PFM was controlled by a digital signal processor (0.5 ms/cycle) to reduce the effect of the PFM's dynamics on the subjects' hand. Detailed descriptions of the PFM and controller have previously been published [23].

#### Experimental Setup

Movements investigated in this study were right-handed forward reaching movements in the horizontal plane at subjects' shoulder level. Movements were made from a 2.5 cm diameter start circle centered 31 cm in front of the shoulder joint to a 3 cm diameter target circle centered 56 cm in front of the shoulder joint. The origin of the coordinate system was centered at the shoulder with the positive *y*-axis corresponding to the straight line from the shoulder joint to the target circle and with the positive *x*-axis corresponding to the line from the shoulder joint to the right direction. The subjects' view of the PFM and arm was blocked by an opaque tabletop positioned above the arm and the PFM. The start and target circles were projected on to the tabletop by an overhead projector throughout all of the experiments. The projector was also used to display a 0.5 cm diameter circular cursor used to track instantaneous hand position when appropriate for the visual conditions as described below. A computer monitor positioned beyond the PFM in front of the subject provided knowledge of results about movement duration (SHORT, LONG, OK) and final hand position (OUT, OK). If a subject received “OK” for all parameters then the trial was considered successful. Movement duration was considered OK within the range 600±100 ms. The movement duration was constrained to be within this range for all parts of the experiment. Subjects' hand position was displayed with the cursor before the start of each trial in order to facilitate movement to the start circle. Once the cursor was moved within the start circle a trial was initiated by three beeps spaced at 500 ms intervals. Subjects were instructed to begin movement on the third beep and to try to reach the target circle by the fourth beep, 600 ms later. Two additional beeps spaced 500 ms apart were heard once the target circle was reached to indicate how long subjects had to hold a steady hand position within the target circle. No instructions were given to the subjects about the trajectory that they should perform in order to complete the task. Subjects were only instructed that they were required to perform successful movements in order to complete the experiment.

#### Force Fields

Learning and stiffness were examined under three different environmental dynamics or force fields. These three environments were: a null force field (NF) (baseline), an unstable position-dependent divergent force field (DF) (Figure 1C) where any deviation from the y-axis was magnified by the negative elastic forces of the DF and a velocity-dependent clockwise curl force field (CF) (Figure 1D). The DF was implemented as
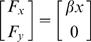
(1)where the force exerted on the hand by the PFM (*F_x_*, *F_y_*) depended on the position of the subjects' hand (*x*) relative to the y-axis. *β* was chosen as 400 N/m or 300 N/m for male and female subjects respectively so that the forces experienced would be relatively strong. These values were chosen based on previous work using this type of force field [10], [24]. During movements in the DF, a safety boundary was implemented such that the force field was removed if the subject deviated more than 5 cm to the right or the left of the y-axis. Due to the destabilizing nature of the DF, the force field was also removed once the subject reached the end target. The CF was described by
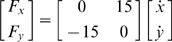
(2)where the force exerted by the PFM was dependent on the subjects' hand velocity (*ẋ*, *ẏ*). This stable force field would produce a stable interaction with the subjects' limb. All subjects experienced each environmental condition under both visual feedback and no visual feedback conditions.

#### Visual Conditions

This experiment was performed to test the effect of visual feedback on learning and final stiffness after adaptation to presentation of novel environmental dynamics. Two feedback conditions, online visual feedback and no online visual feedback, were presented. For online visual feedback, the cursor representing hand position was shown throughout the entire trial. This cursor was a 0.5 cm diameter circular cursor projected directly over top of the subject's hand, tracking the instantaneous hand position. This was updated at a refresh rate of 60 Hz. For the condition of no visual feedback, as soon as the movement was initiated the tracking cursor was turned off for the duration of the movement. Once the final two beeps were heard (1000 ms after the end of the movement) the cursor was displayed again so that subjects could see their final hand position and move the hand back to the start circle to begin the next trial. No information about the actual trajectory was presented to the subjects. The start and target circles were displayed throughout the entire experiment under both visual conditions.

Subjects were randomly divided into two groups (four subjects in each group): vision and no-vision. The no-vision subjects were exposed to each environmental condition under the no visual feedback condition first before experiencing each environmental condition under the visual feedback condition. The vision group was exposed to each environment under the visual feedback condition first and then under the no-vision condition. In both groups the NF field was experienced first before the other two force fields so that subjects could get accustomed to the PFM. The order of presentation of the DF and CF fields was balanced between subjects in each group. This design of the experiment has subjects adapt to the same force fields both with visual feedback and without feedback. One confounding factor in this design is that there could be some retention of what was learned in the first condition which could affect performance on the second condition. While this effect may be partially limited through the random presentation of force fields it could still affect factors such as speed of learning. However, by having subjects adapt under both conditions, direct comparisons of endpoint forces, trajectory variability, and particularly endpoint stiffness, which have large components of individual variability, can be directly compared across the conditions using repeated measures. Subjects performed both the vision and no vision experiments so that the stiffness measurements can be accurately compared across the conditions. This would not be possible using a design where different groups of subjects performed learning under each condition.

#### Learning

Prior to the beginning of the experiment, all subjects trained for one day in the NF in order to adapt to the natural dynamics of the PFM itself as well as accustom them to the task constraints. On these pre-experimental days, full visual feedback of the subjects' trajectory was provided. The experiment was conducted over a period of six days for each subject so that only one of the three force fields under one of the two feedback conditions was ever experienced on a particular day. Each daily experimental session consisted of two parts: learning and stiffness estimation. In the learning phase subjects first performed 30 successful movements in the NF, where a successful movement was considered as one which ended within the allotted time and within the target circle. After 30 successful trials, the force field was activated. Subjects were unaware as to the start of the force field activation as no information was given about the number of trials before the force field would be activated. Subjects were then required to practice in the force field until 100 successful movements were performed. All trials were recorded whether successful or not. The stiffness measurement phase followed the learning phase after a short 5 minute break (see stiffness estimation below). Note that for the NF only the stiffness estimation phase was performed since the learning of novel dynamics was not required in the NF. All subjects had previously trained with the manipulandum in the NF field.

#### Stiffness Estimation

We measured stiffness in the CF and DF force fields after extensive learning, as well as in NF movements. Full details of the stiffness estimation procedure can be found elsewhere [25]. During the stiffness estimation phase of the experiment, subjects were first required to make 20 successful movements in the force field previously learned. This was followed by one hundred and sixty movements, of which eighty were randomly selected for stiffness measurement. For each of the 80 trials in which stiffness was measured, the PFM briefly displaced the subjects' hand by a constant distance at the midpoint of movement in one randomly chosen direction out of the set {0°, 45°, 90°, 135°, 180°, 225°, 270°, 315°}. The displacement moved the subjects hand a fixed distance away from the predicted trajectory using the algorithm of [25]. This displacement had an amplitude of 8 mm and lasted 300 ms. This was composed of a 100 ms ramp away from the current trajectory, a 100 ms hold portion, and a 100 ms ramp back towards the predicted trajectory. During the hold phase of the perturbation, the hand was displaced with the predicted velocity of the unperturbed movement. Assuming that the perturbation is perfect there would be no difference in velocity between the perturbed and unperturbed trajectories, eliminating any contribution of damping to the change in measured endpoint force. Although the prediction is not perfect, our results indicate that the errors are small and that the average prediction over several trials is close to the average of the actual trajectory [25]. Therefore we can be confident that the forces due to damping did not introduce error in the stiffness estimates. The average restoring force and position displacement measured at the subjects' hand during a 50 ms interval in the final half of the hold period of the perturbation was used to estimate endpoint stiffness (from 140ms until 190ms after the perturbation onset). This interval was chosen to avoid contamination of the stiffness estimates from large forces due to inertial properties of the limb during the ramp portions of the displacement. In the stiffness estimation trials the force field was on both before and after the displacement.

### Data Analysis

#### Learning

Learning in the different force fields was calculated as the error relative to the straight line joining the start and end targets (the *y*-axis). This measure of learning was used since movements in the NF as well as movements after adaptation in the force fields tended to be relatively straight. The absolute hand path error was
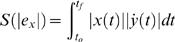
(3)and represents the total area between the movement path and the straight line joining the start and end targets. Thus small hand path error values denote relatively straight trajectories. *x* (*t*) and *ẏ* (*t*) represent the shoulder-joint centered Cartesian x-position and y-velocity of subjects' hand respectively, while *t_o_* was taken 300 ms prior to the y-velocity crossing a threshold of 0.05 m/s and *t_f_* represents the time of movement termination (time when curvature exceeded 0.07 mm^−1^). To take into account the effect of switching off the DF once subjects crossed the 5 cm safety boundary, movements were assumed to remain at the boundary for the duration of the movement from the time it was crossed for calculation of the handpath error. To test whether learning occurred, a separate ANOVA was performed for each field and visual condition to compare the absolute hand path error on the first and last 10 trials of learning. Each ANOVA had a main effect of Learning (early = first ten trials; late = last ten trials), and random effect Subjects.

In order to compare learning, exponential curves were then least-square fitted to the hand path errors for each subject in order to compare learning rates as a function of trial number (using Matlab® R14 function lsqcurvefit). The exponential curve took the form of
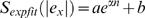
(4)where *a* is the initial or before effect error caused by the onset of the force field, *α* is the learning rate, *n* is the trial number and *b* is the steady-state error after adaptation. In order to examine the rate of learning as obtained from the best fit exponential function (Equation 4), an ANOVA with main effects of Vision (Visual Condition), Field (type of force field), and Order (order of learning), and random effect Subjects was performed on the data. Second level interaction terms were also examined for (Field*Vision) and (Field*Order). Separate ANOVAs for the CF learning rates and for the DF learning rates were performed in order to further examine the Order effect in the two fields. The ANOVA had main effects of Vision (visual condition) and Order (order of learning), and random effect of Subjects.

The speed of learning across the force fields and visual conditions was also examined by looking at the number of trials required in order to complete 100 successful trials. A separate non parametric Kruskal-Wallis test was performed on the results of each force field with grouping variable Vision (visual condition).

#### Trajectories

The linearity ratio [26] was used as a measure of the lateral deviation of movement trajectories. It was calculated as *l* = *a*/*b*, where *a* is the maximum orthogonal deviation of the trajectory from the straight line joining the start and end targets and *b* is the distance of this line. The linearity ratio was determined using the mean trajectory of the last 20 successful trials of learning averaged across all subjects for each field and visual condition. The linearity ratio was calculated in order to examine differences in trajectories between the vision and no vision conditions in each field as in [27], in which they used this measure, but only for free movements without external perturbing forces. Differences between visual conditions for each force field were tested for significance using the non-parametric Kruskal-Wallis test.

When making repetitive movements there is always variability in the individual trajectories. In order to examine differences in the variability of these movements across the conditions, the mean standard deviation of movement trajectories in the *x*-direction as a function of *y*-position was also calculated. This measure, independent of the actual trajectory chosen, was estimated across all subjects for each field and visual condition for the last 20 learning trials whether successful or not. However, to prevent single trials from unduly influencing the results, selected outlying trials were removed from the data if their path was far outside the trajectories of the rest of the trials. The criterion for removal was if the trial trajectory went outside 2.5 * the standard deviation of the twenty trials. If trials were removed then the next trial was included such that a total of 20 trials were used for each subject in each force field. In the NF field, 4 trials were removed out of the 320 trials total (vision: 1 trial; no vision 3 trials). In the CF, 25 trials out of the 320 trials were removed (vision: 14 trials; no vision 11 trials). In the DF, any trials which exited the safety zone were removed automatically. Out of the trials remaining, 6 trials out of the 320 trials were removed (vision: 3 trials; no vision 3 trials). The y-position data was transformed to a percentage of the maximum *y*-position of each individual trial so that all movements were aligned on the same scale since each movement terminated at a slightly different position. The corresponding *x*-position value in Cartesian space was found at each 1% increment of the y-position with the *y*-position ranging from 0–100% of the total movement. Variability was then calculated in the form of standard deviation for each individual subject and then averaged across all subjects. The movement variability was examined in order to determine the effects of vision. Significant differences between the standard deviation in the visual and non-visual feedback conditions were examined using paired t-tests with Bonferroni correction for multiple comparisons. At each 1% of movement distance from 2% to 98% of the movement distance, five points (±2%) were included in the paired t-test. Significant differences were considered at the 0.05 and 0.001 levels.

#### Endpoint Forces

Endpoint forces (measured at the handle of the manipulandum) from the last 20 successful trials during the learning phase of the experiment for the CF and DF were analyzed. The last 10 successful trials were used in order to look at endpoint forces after adaptation to the novel dynamics. Since there was no learning phase for the NF, the last 10 successful pre-trials during the stiffness estimation phase of the experiment for the NF were used instead. Mean endpoint forces for each subject at the mid-point of movement (the 50 ms stiffness estimation time interval) were examined to determine if subjects adapted to the force fields with or without vision using different endpoint forces. The time interval of stiffness estimation was chosen for this comparison so that it would be possible to examine whether any differences in stiffness between the visual conditions could have be explained by changes in endpoint forces. An ANOVA with main effect of conditions (6 levels = 3 fields×2 visual conditions), and random effects of subjects was used to examine this. If a significant main effect of conditions was found then a post-hoc test (Tukey's HSD) was used to test for significant differences across the conditions.

#### Endpoint Stiffness Estimation

Using the force and position data recorded during the stiffness estimation phase of the experiment, the 2×2 endpoint stiffness matrix **K** was estimated for each subject and condition. Endpoint stiffness was estimated by performing a linear regression on the mean change in endpoint force (Δ*F_x_*, Δ*F_y_*) and the mean change in position (Δ*x*, Δ*y*) during the 50 ms interval in the last half of the hold phase of the perturbation window. The relationship is shown in the equation: 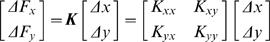
(5)Endpoint stiffness was then represented by plotting stiffness ellipses which show the elastic force produced per unit displacement [28] using the singular value decomposition method [29] to estimate the size, shape and orientation of the endpoint stiffness ellipses. For the size, shape and orientation of the stiffness ellipse, and each element of the stiffness matrix (*K_xx_*, *K_xy_*, *K_yx_*, *K_yy_*) an ANOVA was performed with main effect condition (6 levels = 2 visual conditions×3 force fields) and random effect Subjects. If a main effect of condition was found to be significant, differences in stiffness between the visual feedback and no visual feedback conditions were examined using post-hoc tests (LSD).

In order to examine whether stiffness was primarily increased in the *x-* or *y-*axes after adaptation to the DF, the ratio of the stiffness increase in the *K_xx_* and *K_yy_* terms between the DF and NF fields was calculated. The relative increase in the *K_xx_* term was compared to the relative increase in the *K_yy_* term and tested with a paired t-test. This was performed separately for both the visual feedback condition and the no-visual feedback condition. For the NF condition, the mean of the visual and non-visual conditions was used.

#### Statistical Testing

Statistical analysis was performed using SPSS 10.0 (SPSS, Chicago, IL) with the exception of the multiple paired t-tests examining the variability of movements which were performed in Matlab® R14. Statistical significance was considered at the 0.05 level for all statistical tests. ANOVAs were examined in SPSS using the general linear model. When post-hoc tests were performed after a significant main effect was found, Tukey HSD test was used in all cases except for the testing of stiffness. For the testing of stiffness, the more liberal Fisher LSD post-hoc test was used. LSD was chosen, rather than a more conservative test such as Tukey's HSD, because it is more robust to a Type II error. In the case of stiffness results it was important to avoid cases where no difference between the two visual conditions was reported when there was a real difference between the conditions. The data was examined to determine if it came from a normal distribution using either the Kolmogorov-Smirnov test with Lilliefors significance correction or the Shapiro-Wilk test, depending on the degrees of freedom. The data was also visually inspected to confirm that it did not appear to depart from a normal distribution with homogeneous variances. Most data sets appeared to be from a normal distribution allowing for the use of parametric statistics. However, both the linearity ratios data set and numbers of trials required to achieve 100 successful trials data set were tested with the non-parametric Kruskal-Wallis test.

## Results

### Learning

**Figure 2:**
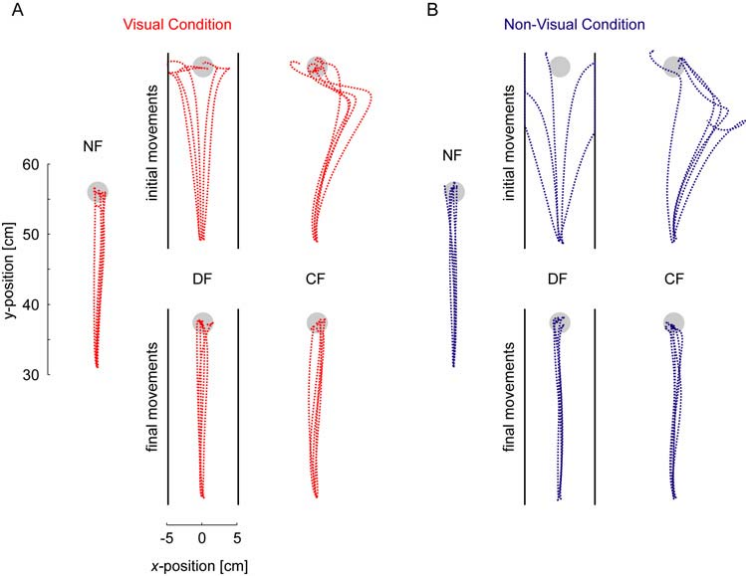
Initial and final movement trajectories. (A) Initial and final trajectories in the NF, DF and CF with visual feedback. (B) Initial and final trajectories in the NF, DF and CF without visual feedback. The first 5 movements and the last 5 movements during learning for subject 1 are shown. The black lines represent the 5 cm safety boundary implemented in the DF at which point the force field was turned off.

Subjects initially made movements in the null field condition either with or without vision. Under these conditions, all subjects were able to make roughly straight movements of appropriate speed to the target. When the force fields were unexpectedly applied, subjects' trajectories were disturbed by the change in forces experienced. Gradually subjects were able to reduce the disturbance created by the novel dynamics by learning to compensate for it. Figure 2A depicts initial and final movement trajectories in the NF, DF and CF with visual feedback and Figure 2B shows initial and final trajectories in the same fields without visual feedback. The relatively straight trajectories typical of point-to-point reaching movements are seen in the initial as well as the final movements in the NF for both visual conditions. Initial movements in the DF were either perturbed to the right or the left of the y-axis depending on the initial motor output variability. This movement variability was amplified by the negative elastic forces of the DF, magnifying any initial deviation from the y-axis. However subjects gradually straightened out their movements. In the CF, movements were initially perturbed to the right, but again subjects were able to adapt to the imposed dynamics and were able to make movements similar to those in the NF after learning.

**Figure 3:**
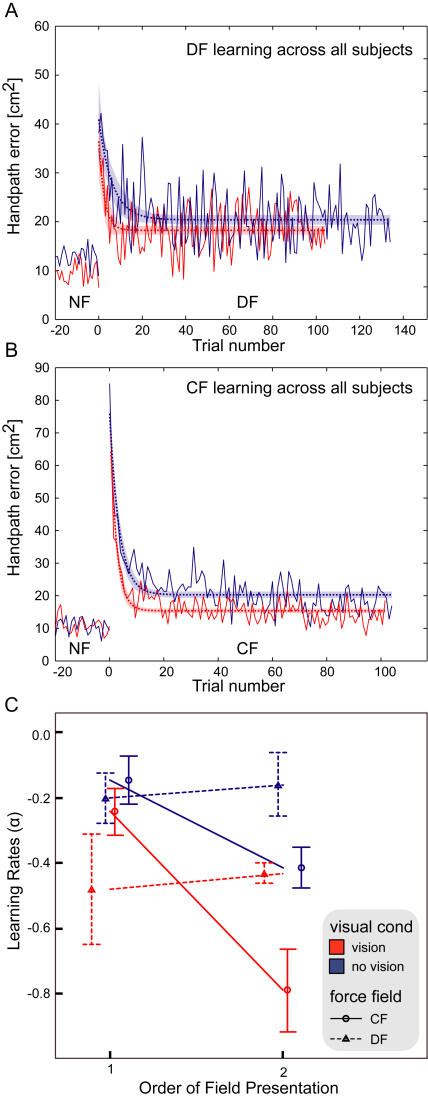
Mean learning represented by absolute hand-path error averaged across all subjects. (A) Mean learning in the divergent force field (DF) is shown using thick solid lines. Learning with vision is shown in red, while learning with no vision of the endpoint position is shown in blue. The thin dotted line and shaded region illustrates the least-squares fitted exponential curves with 95% confidence intervals. Data occurring before 0 trials was performed in the null force field (NF) colored according to the visual conditions as above. (B) Mean learning in the curl force field (CF). (C) A comparison of learning rates (α) across the conditions plotted against the order of subject performance. The learning rate was estimated for each subject in each condition separately using equation (4). Red and blue lines indicate the vision and no-vision conditions respectively. The DF field is plotted with dotted lines whereas the CF field is plotted with solid lines. Order = 1 indicates that this condition (vision or no-vision) was performed before learning the same field with the other visual condition, whereas order = 2 indicates the opposite. Error bars represent standard error of the mean.

To further confirm that learning occurred under both visual conditions in the force fields we examined the handpath error. The handpath error is a measure of the area between the actual trajectory and the straight-line path joining the start and end targets. A clear learning effect across all subjects is seen by examining the mean absolute hand-path error for the visual feedback and no visual feedback groups in the DF and the CF (Figure 3). At the initial stage of learning when compared with hand-path error in the NF trials, large errors were induced by the force fields. However subjects were able to gradually reduce errors to a steady level in both force fields and under both visual conditions. In the DF, under both vision and no-vision conditions, the error was significantly reduced from the first ten trials to the last ten (vision: *F*_(1,151)_ = 4.987; *p* = 0.027) (no-vision: *F*_(1,151)_ = 15.577; *p*<0.001). Similarly in the CF the last ten trials had lower handpath errors both with vision (*F*_(1,151)_ = 50.57; *p*<0.001) and without vision (*F*_(1,151)_ = 104.28; *p*<0.001). Subjects were able to adapt to or learn both stable and unstable dynamics regardless of whether visual feedback was given or not.

As learning occurred under all conditions, we next examined the speed of learning. In order to compare learning speeds under the various conditions, both the learning rate and the total number of trials required for success were examined. The learning rates were obtained by least squares fitting of the exponential curves to the handpath error data for each subject. The learning rates across all conditions were tested with an ANOVA. Significant main effects were found for Vision (*F*_(1,19)_ = 18.719, *p*<0.001) and Order (*F*_(1,19)_ = 9.368, *p* = 0.006) but not for Field (*F*_(1,19)_ = 1.855, *p* = 0.189). A significant interaction between Field and Order was also obtained (*F*_(1,19)_ = 14.709, *p* = 0.001), whereas the interaction between Field and Vision was not significant (*F*_(1,19)_ = 0.109, *p* = 0.745). The learning rates are shown in Figure 3C. The statistical results indicate that overall (across the two force fields) subjects learned faster with visual feedback compared to when visual feedback was not present. The significant order effect indicates that subjects were faster the second time that they adapted to the force field compared to the first. However, this is complicated by the significant interaction effect between Field and Order. It can be seen in Figure 3C that the order of learning only affected the rate of learning in the CF force field and not the DF field. This was further confirmed by performing separate ANOVA's on the CF and DF data. In the DF there was no significant Order effect (*F*_(1,6)_ = 0.548, *p* = 0.487) whereas in the CF there was a significant Order effect (*F*_(1,6)_ = 23.479, *p* = 0.003). These results indicate that subjects were able to reduce the handpath error quicker the second time they adapted to the CF field whether this was with or without visual feedback. This was not true of the adaptation to the DF where there was no significant change in the learning rate the second time subjects adapted to the field.

Different numbers of trials were required in order to complete 100 successful trials under the various conditions. The mean trials across subjects (±standard deviation) were: DF vision = 134.3±34.8; DF no vision = 191.4±82.5; CF vision = 107.0±3.5; CF no vision = 159.8±31.5. There were significantly fewer trials required with visual feedback in both the DF (Kruskal-Wallis, *Chi-Square*_(1)_ = 3.982; *p* = 0.046) and CF (Kruskal-Wallis, *Chi-Square*_(1)_ = 7.467; *p* = 0.006) force fields.

### Trajectories

Although adaptation occurred under both the vision and no-vision conditions, upon closer examination of movement trajectories, some differences in the kinematic features were uncovered. The mean trajectory used to reach the target location was examined with the linearity ratio. The ratio was found to be slightly larger for the no vision condition in both the NF and the CF, indicating that the movements were more curved under the no-visual condition in these two force fields. In the NF, linearity averaged across all subjects was 0.0285±0.0126 and 0.0332±0.0154 for the visual and no visual conditions respectively. In the CF linearity was 0.0405±0.0197 with vision and 0.0488±0.0228 without vision. In both force fields, the movements in the visual condition were significantly more linear than those in the non-visual condition (NF: Kruskal-Wallis, *Chi-Square*_(1)_ = 7.329; *p* = 0.007) condition (CF: Kruskal-Wallis, *Chi-Square*_(1)_ = 12.687; *p*<0.001). These results are consistent with previous findings [27], [30] for mid-line horizontal reaching movements in the sagittal direction. However movements in the DF did not exhibit significant differences in linearity between the two visual conditions (0.0470±0.0223 with vision and 0.0478±0.0241 without vision) (Kruskal-Wallis, *Chi-Square*_(1)_ = 0.013; *p* = 0.911).

**Figure 4:**
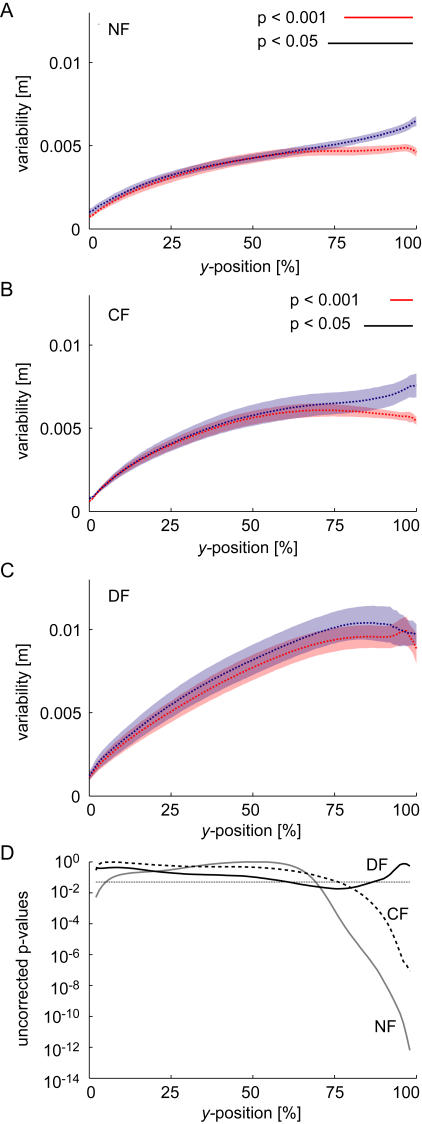
Mean movement variability in the x-direction as a function of the percentage of total movement distance in the *y-*direction shown for the final 20 trials of learning averaged across all subjects. (A) For movements in the NF. The mean value (dotted line) plus mean standard error (shaded areas) are shown for both vision and no-vision conditions. While the variability is higher in the no-vision condition throughout the movement this becomes most prominent towards the end of the movement. Times with a significant difference between the visual and non-visual conditions are indicated by the black (*p*<0.05) and red (*p*<0.001) lines at the top of the figure (Bonferroni corrected for multiple comparisons). (B) Movements in the CF. The largest difference in the variability between the no-vision and vision conditions occurs at the end of the movement. (C) Movements in the DF. No significant differences were found. (D) Uncorrected *p*-values for the paired t-tests performed for each of the three force fields. The uncorrected *p* = 0.05 level is indicated with the thin dotted line.

While not essential for learning to occur, visual information can be very useful during a movement to correct for errors and ensure movements reach the target. In order to examine whether there is evidence for this during movements a measure of the positional variability about the mean movement under each condition was calculated. In particular we calculated the standard deviation in the *x*-direction of twenty movements as a function of the percentage of the total movement distance in the *y*-direction (Figure 4). This measure is independent of the trajectory used to get to the target; it only considers the variability about the mean trajectory. The variability of movements is shown to gradually increase with the percentage of distance traveled in the *y*-direction in the NF, CF and DF under both visual conditions. However in the case where online visual feedback is available the movement variability during the last portion of the movement tends to decrease in the NF and CF. On the other hand, when subjects are deprived of vision during movement, this reduction in variability near the end of movement is not observed. Significant differences between the visual and non-visual conditions were found only at the end of the movements. A significant difference in the variability during movements in the NF started at 73% of the movement distance, whereas in the CF this difference started at 84% of the total movement distance. This suggests that online visual feedback was used by subjects to reach the 2.5 cm end target with greater accuracy but that in general subjects did not use this information until near the end of the movement. In the DF, there were no significant differences at the 0.05 level found at any point during the movements. In contrast to the NF and CF force fields, no differences were seen towards the ends of the movements. In the DF, trials which move too far from the straight-line of zero force are more likely to be pulled by the force field to outside the safety zone. This likely occurs for trials both with and without visual feedback creating little difference in the endpoint variability between the two groups.

### Endpoint Force

**Figure 5:**
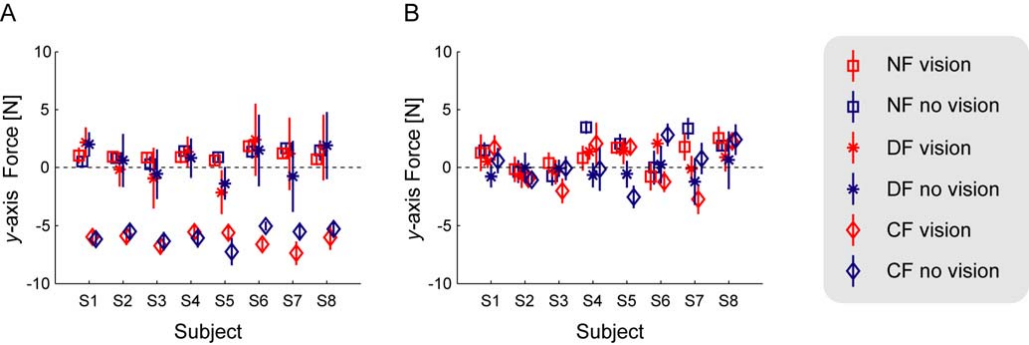
Endpoint Force exerted against the handle of the manipulandum after learning. (A) The mean endpoint force in the *x-* axis across all eight subjects during the 50ms period in the middle of the movement over which the endpoint stiffness is estimated. The visual conditions are shown in red, and the non-visual conditions in blue. The NF is shown with the square, the DF with the asterisk, and the CF with the diamond. The error bars indicate the standard deviation of the force across the ten trials. (B) Mean *y-*axis endpoint force across the conditions for each subject.

After adaptation to the force fields, the endpoint force that the subjects produced against the robotic interface was examined (Figure 5). Similar levels of force were produced between the visual conditions for each force field. As expected, the force in the CF field was larger in the x-axis. The endpoint force in the DF and NF fields were similar, close to zero in the x-axis. The last ten trials in the learning phase were used to test if the forces were significantly different across the conditions. The mean endpoint force was determined for the 50ms interval during which the stiffness was estimated. In the *x-*axis, there was a significant main effect for condition (*F*_(5,35)_ = 157.0, *p*<0.001). Using Tukey's HSD post-hoc test, it was determined that there were two distinct groups: the two CF conditions (vision and no-vision) were significantly different than the other four conditions (DF and NF) (*p*<0.001 for all comparisons). The two CF conditions were not significantly different (*p* = 0.71). Similarly there were no significant differences across the visual conditions in either the DF (*p* = 0.97) or NF fields (*p* = 0.75). None of the other possible comparisons were significantly different either (*p* = 0.257, *p* = 0.654, *p* = 0.188). There was also no significant main effect for the conditions in the y-axis (*F*_(5,35)_ = 1.25, *p* = 0.31).

### Stiffness

**Figure 6:**
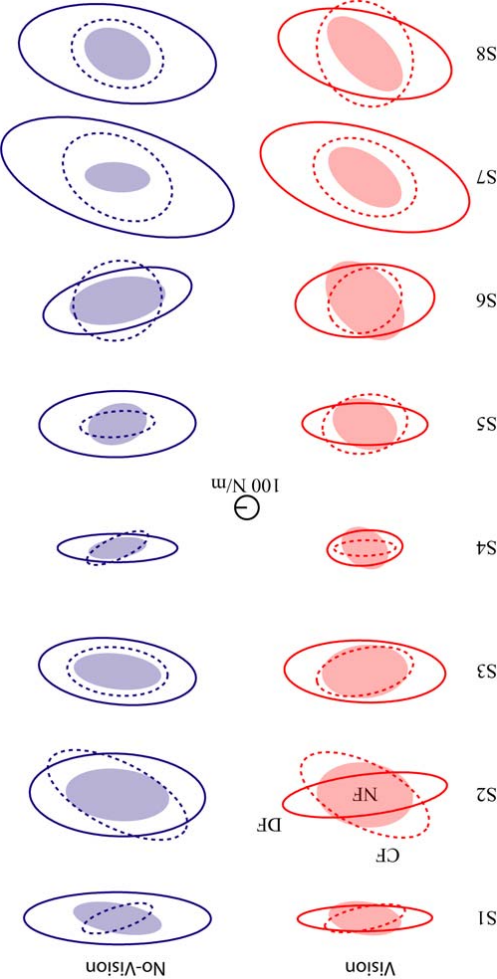
Endpoint Stiffness for all eight subjects (S1-S8) under all conditions represented as an ellipse. Visual conditions are shown in red while non-visual conditions are shown in blue. The NF stiffness ellipse is shown with the light filled ellipse, the DF stiffness ellipse is shown with the solid line, and the CF stiffness ellipse is shown with the dashed line. The stiffness of the limb is measured in the middle of the movement.

After learning was finished in each condition, subjects' endpoint stiffness was estimated using controlled position perturbations [25]. The endpoint stiffness was estimated at the mid-point of the reaching movement under all conditions (Figure 6). While each subject exhibited large individual differences in terms of shape and size of the endpoint stiffness ellipse, each subject also showed quite similar results between the vision and non-vision conditions in each field. The variability between the visual and non-visual conditions was much less than the individual variance between subjects.

**Figure 7:**
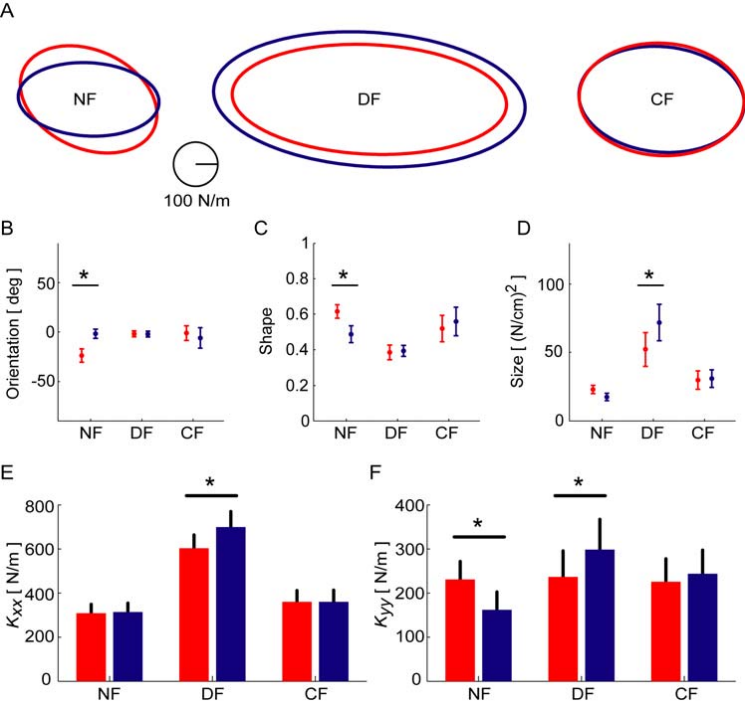
Changes in the endpoint stiffness after learning under the visual (red) and non-visual (blue) conditions. (A) Ellipses averaged across all subjects for the NF, DF and CF. (B) The mean endpoint stiffness ellipse orientation across the six conditions. (C) The mean endpoint stiffness ellipse shape. (D) The mean endpoint stiffness size in the NF, DF and CF. (E) The mean *K_xx_* stiffness in the NF, DF and CF. (F) The mean *K_yy_* stiffness across the three conditions. The symbol * indicates that there is a significant difference at the *p* = 0.05 level. For all figures, error bars represent the standard error of the mean.

To determine the overall effect of vision on the endpoint stiffness of the limb the mean endpoint stiffness ellipses averaged across all subjects for the three different dynamics and under the vision and no vision conditions were examined (Figure 7). Differences in the orientation, shape and size of the endpoint stiffness ellipses were examined. After significant main effects of force field were found for orientation (*F*_(5,35)_ = 2.907, *p* = 0.027), shape (*F*_(5,35)_ = 4.274, *p* = 0.004), and size (*F*_(5,35)_ = 11.054, *p*<0.001), post-hoc tests (LSD) were used to examine differences between the visual conditions. While differences occurred between the three force fields, this modification of stiffness has been extensively examined in previous work [16] and will not be covered here. The results therefore focus on the differences between visual conditions for a single force field by reporting the appropriate post-hoc comparisons. In the NF, there was a significant difference in the orientation between the visual and non-visual conditions (*p* = 0.005). Similarly, there was a significant difference in the shape between the visual and non-visual conditions (*p* = 0.048) but not in the size (*p* = 0.525). In the DF, the endpoint stiffness was significantly increased in size (*p* = 0.031) for the no vision condition compared to the visual condition. However, there were no significant differences in either the orientation (*p* = 0.969) or shape (*p* = 0.891) between the visual and non-visual endpoint stiffness. Finally in the CF, there were no differences in the size (*p* = 0.898), shape (*p* = 0.526) or orientation (*p* = 0.506) of the endpoint stiffness ellipse between the visual and non-visual conditions.

The four elements of the stiffness matrix were also examined for confirmation of the previous results. A significant main effect for both the *K_xx_* term (*F*_(5,35)_ = 29.909, *p*<0.001) and *K_yy_* term (*F*_(5,35)_ = 4.697, *p*<0.001) were found. There were no significant main effects found for the *K_xy_* (*F*_(5,35)_ = 1.555, *p* = 0.198) and *K_yx_* (*F*_(5,35)_ = 0.599, *p* = 0.701) terms of the stiffness matrix. The differences in the *K_xx_* and *K_yy_* terms were investigated further with post-hoc tests. There were no significant differences between the visual conditions in the NF force field (*p* = 0.903) or the CF force field (*p* = 0.998). However the *K_xx_* term was significantly larger in the no-vision condition compared to the vision condition in the DF (*p* = 0.033). There was also no significant difference in the *K_yy_* term between the visual conditions in the CF (*p* = 0.530).There were however significant differences in the *K_yy_* term for both the NF and DF force fields. In the NF, the *K_yy_* in the visual condition was larger than that in the no-vision condition (*p* = 0.021) whereas in the DF, the *K_yy_* term in the no-vision condition was larger than that in the visual condition (*p* = 0.036).

In the NF, there were small differences in between the visual conditions. No significant difference was found in the *K_xx_*-component of stiffness, however a significant difference was found in the *K_yy_*-component of endpoint stiffness between the vision and no vision conditions. This occurred despite similar levels of endpoint forces for both visual conditions in the NF. When the stiffness ellipses are examined for the individual subjects (Figure 6), it can be seen that only subjects S4, S6 and S7 show this large change in orientation between the visual and non-visual conditions. It is therefore not clear whether this difference in stiffness is a true representation of the effect of moving under different visual conditions or other possible factors such as speed of movement.

After adaptation to the stable velocity-dependent CF there were no significant differences for any of the endpoint stiffness characteristics between the two visual conditions. Subjects adapted using similar levels of endpoint force and produced similar changes in the endpoint stiffness whether visual feedback was present or not for adaptation.

As observed in previous studies, stiffness ellipses in the DF were mainly increased towards the direction of instability without any changes in endpoint force. The selective increase in x-stiffness was seen under both visual feedback conditions which can be interpreted to mean that subjects adapted their endpoint impedance to attain stability in the DF in a similar manner whether or not online visual feedback was available. No significant differences were found between the two visual conditions for the ellipse orientation or shape. However there was a significant difference in the size of the stiffness ellipses between the two visual conditions. Examination of the stiffness matrices demonstrated that the no-visual condition was larger in both the *K_xx_* and *K_yy_* terms. This indicates that the stiffness was increased both in the *x-* and *y-*axes. In order to confirm that the stiffness was still primarily increased in the direction of the instability of the environment, the ratio of the stiffness increases in the two stiffness terms were compared. In the visual condition, the *K_xx_* stiffness in the DF was on average 1.95 times the size of the *K_xx_* stiffness in the NF whereas the *K_yy_* was 1.20 times the size in the NF. The relative increase of the *K_xx_* stiffness term was larger than the *K_yy_* stiffness term (paired t-test, *t*_(7)_ = 6.767, *p*<0.001) indicating that the NF stiffness was not simply scaled up equally in the *x-* and *y-*axes. In the non-visual condition the *K_xx_* stiffness after adaptation to the DF was 2.30 times the size in the NF and for the *K_yy_* term it was 1.53 times the size. Similarly this increase was significantly larger in *K_xx_* than in *K_yy_* (paired t-test, *t*_(7)_ = 4.982, *p* = 0.002). Therefore, the relative increase in the stiffness after adaptation to the DF occurred primarily in the *x-*axis for both the visual and non-visual conditions. This indicates that the stiffness had still been selectively increased in the direction of the instability of the force field.

## Discussion

Eight subjects performed movements in three environments: a null field (NF), a stable force field (CF) and an unstable force field (DF), under two different visual conditions. Whether subjects were either presented with or without online visual feedback of their hand trajectory, they learned to compensate for both unstable and stable dynamics in order to make smooth movements to the target. Similar levels of endpoint force and endpoint stiffness for both visual feedback and non-visual feedback conditions were seen after adaptation to the two force fields. These results indicate that whether subjects were presented with visual feedback or not, the subjects adapted to the force fields in the same manner: learning the appropriate level of joint torques and endpoint stiffness for the environment. One distinct difference between the vision and no-vision condition was that the endpoint stiffness ellipse was slightly larger after adaptation to the DF with no visual feedback. However in both visual conditions *K_xx_* (the stiffness in the *x-*direction produced by a perturbation in the *x-*direction) increased by a greater percentage than *K_yy_* (the stiffness in the *y-*direction produced by a perturbation in the *y-*direction). This indicates that under both conditions the endpoint stiffness was increased primarily in the direction of the instability. Under the visual feedback condition subjects were able to reduce their handpath error more quickly. They also tended to make straighter movements when in either the NF or CF fields. In the same two fields, the visual movements also exhibited less variability than the non-visual trajectories but only towards the end of the movements.

### Visual Feedback is not necessary for Learning Dynamics

Visual feedback of the trajectory is not required for adaptation to stable dynamics, nor is it required for adaptation to unstable dynamics. Subjects were able to adapt to the force fields both with and without visual feedback of their trajectory. Previous work has shown that subjects do not rely on visual feedback for learning stable dynamics [18]–[21]. While DiZio and colleagues [21] demonstrated that congenitally blind individuals are able to adapt to the perturbing effects of a Coriolis force field produced by a rotated room, the other three studies demonstrated that fully sighted individuals are able to adapt to novel robotic force fields with varying degrees of visual feedback. It was demonstrated that when subjects were presented with delayed visual trajectory information during adaptation to curl force fields, they were still able to reduce their kinematic error and make straight reaching movements [18]. It was also shown that similar reductions in kinematic error were produced when subjects received only delayed feedback about the final location of their hand [20]. One interesting effect was found when subjects were presented with only visual information about the extent of their movement with no information about the errors perpendicular to the movement direction [19]. In this case, subjects were able to straighten out the movements and adapt to the perturbing effects of the novel field, however they did not compensate for the change in direction produced by the original impulse to the force field and therefore did not reach towards the original targets. The general finding of these previous studies, that online visual feedback of hand location is not required for adaptation to novel dynamics, is confirmed by our results and extended to the unstable dynamics condition, despite that finding that such dynamics are significantly more difficult to learn [12]. Subjects were able to reduce the handpath error, and make smooth straight movements to the target. In the non-visual feedback condition, vision of the final hand location was only provided one full second after the movement had been finished. No correction to the target was possible after the feedback was given, and the force field was always turned off at this point in time. This off-line visual feedback therefore provided only the final error amount and direction, and could not be used to adapt to the dynamics along the trajectory. In contrast with some previous studies [18], no information on the trajectory was provided after the subject had finished making the movement. Visual feedback of the trajectory is therefore not required for adaptation to novel dynamics. This result is not unexpected as congenitally blind individuals are able to walk and use tools (two examples of adaptation to unstable dynamics), and have previously been shown to adapt to stable novel dynamics [21]. Clearly, the visual feedback signal is therefore not critical for the dynamics adaptation process.

One interesting finding with the speed of adaptation data was the analysis of the order effects. In particular when the subjects had previously learned the CF with either of the visual conditions, the speed of adaptation was increased for the second adaptation with the opposite visual condition. On the other hand, no such effect was found for adaptation to the unstable DF. When subjects had previously learned the field with one visual condition or not, the speed of adaptation was not affected. One possible interpretation of these results is that previous learning of unstable dynamics does not assist with the re-learning of the same dynamics. This is in contrast with the learning of stable dynamics, where the previous learning of similar dynamics can either assist or hinder the learning of the current dynamics depending on the similarity of the force fields [31]–[33].

### Final Adaptation was similar both with and without Visual Feedback

While it is clear that the subjects could adapt to the novel dynamics both with and without visual feedback, the larger question is whether the adaptation occurs in the same way. It is possible to imagine that if different learning signals were used for adaptation with or without visual feedback, that the end control mechanism (or internal model) could be different. We tested this by examining both the endpoint force and limb stiffness. If subjects used different methods of control with and without visual feedback, leading to different patterns of muscle activation, this would be seen both in the force trace and the endpoint stiffness. For example, if subjects modified the level of muscle co-activation, then this would have changed the shape and/or size of the endpoint stiffness ellipse, whereas a change in the reciprocal activation would have modified both the endpoint stiffness ellipse and the endpoint force. However, if these two measurements are similar both with and without vision, then it would indicate that subjects adapted in a similar style both with and without visual feedback. Previous work examining the adaptation to stable dynamics has not tested whether the adaptation occurred in the same way [18]–[20]. In the CF, subjects produced the endpoint forces which were almost identical both with and without vision. When the endpoint stiffness was measured, there were no significant differences in any of the measures of endpoint stiffness (shape, orientation or size) or within the stiffness matrix itself. The mean stiffness across subjects, both with and without visual feedback, overlay almost perfectly (Fig. 7). This indicates that subjects do not adapt to stable dynamics with increased stiffness when no visual feedback is given. Therefore we have extended the previous work [20] to demonstrate that subjects both with and without vision adapt in the same manner to the novel stable dynamics.

After adaptation to the unstable DF dynamics, subjects again show similar levels of endpoint force, both with and without vision. In the *y-*axis, the same pattern of endpoint force is used to accelerate the arm and produce the movement. In the *x-*axis, the endpoint force is maintained close to zero throughout the movement. Similarly, subjects both with and without vision produced roughly straight movements with no difference in the amount of linearity across the conditions. When the endpoint stiffness was examined there were some similarities and some differences in the endpoint stiffness between the two visual conditions. In both the visual and non-visual conditions, the relative increase in the stiffness after adaptation to the DF occurred primarily in the *x-*axis (increase in *K_xx_* was larger than the increase in *K_yy_*). This indicates that the stiffness had still been selectively increased in the direction of the force field. However the endpoint stiffness was larger when subjects were moving in the unstable environment with no visual feedback. Why would subjects tend to increase their endpoint stiffness in this unstable environment when no visual feedback was present? One possibility is that this arises from increased uncertainty about the environment. The brain needs to estimate the current state of the arm in order to compensate accurately for any small errors in the movement. However, the delayed and noisy sensory feedback and signal dependent noise in the generated motor command produce uncertainty in the brain's estimate of the current state [34]. During normal movements with both visual and proprioceptive feedback, these two sensory information modalities are combined by the brain in order to accurately estimate the current state of the limb. However, when one of these sensory modalities has been removed, there will be increased uncertainty about the current state. In particular, we have removed the visual feedback during forward reaching movements, in fact removing it where it is most sensitive to errors (in the lateral direction) which are now enhanced by the environment [3]. In order to deal with this, the CNS increased the stiffness of the limb to resist the environment instability and reduce the deviation of the position. The increased stiffness will reduce this deviation because increased muscle stiffness attenuates the effect of the motor noise [35]. This will allow the hand to remain closer to the straight line between the two targets, reducing the influence of the force field.

This increased uncertainty about the current state would occur in both the stable and unstable environments without visual feedback, so why did the subjects only increase their limb stiffness in the absence of visual feedback (compared to the condition with visual feedback) in the unstable environment? The answer to this is related to the difference between stable and unstable environments (Fig 1 C,D). In the stable environment, small differences in the trajectories (produced by for example motor noise), result in almost identical forces applied to the hand. Therefore, even if there is more uncertainty about the exact state of the hand, the CNS does not need to compensate for this. The same pattern of motor commands is able to produce the correct movement despite small differences in the trajectory. On the other hand, in an unstable environment, small differences in the trajectory produce large differences in the forces exerted to the hand. The ability to accurately predict where the hand will be at a future time is better in the CF than the DF because of the effect of motor noise coupled with environmental instability [36]. Motor noise results in unpredictably of future position in the DF because the direction of initial deviation cannot be accurately predicted, even with perfect sensory information. In order to compensate for this increased uncertainty of the current state and unpredictability of the future states (trajectory, endpoint forces) in the DF, the subjects increased the stiffness of the limb. The increased stiffness will reduce this deviation because increased muscle stiffness attenuates the effect of the motor noise [35]. This will allow the hand to remain closer to the straight line between the two targets, reducing the influence of the force field.

It is not clear, however, why the increase in stiffness in the no visual feedback condition (relative to the visual feedback condition) occurred in both the *x-* and *y-*axes rather than only in the direction of the instability. One possible explanation is due to the problem with determining the appropriate orientation of the stiffness ellipse with respect to the hand. Depending on the position of the hand, the orientation of the hand, and therefore the orientation of the endpoint stiffness of the hand, relative to the environment would change. If the removal of visual feedback reduces the accuracy of the prediction of the future state of the subject's hand during movement, the exact orientation of the stiffness ellipse appropriate to counteract the instability in the environment may not be able to be predicted. The subjects may increase the stiffness in both the *x-* and *y-*axes in order to produce the appropriate level of stiffness for a variety of likely arm configurations. This possibility could be tested by adding one condition where the subjects only received visual feedback about the extent of their movements, comparable to the work of [19]. Under this condition, subjects would have accurate information about the orientation of their limb, so changes in the endpoint stiffness would be expected only in the x-axis.

### Effect of Visual Feedback on Trajectory

In a previous kinematic study, a statistically significant increase in hand-path curvature was found when subjects' vision was removed using a blindfold as compared to the case when visual feedback was available [27]. Our study also found that movements in both a null force field and after adaptation to a curl force field were straighter with visual feedback of the trajectory than without. However, this effect was not seen in the unstable environment. This is not surprising as straightness is not required to make movements in the NF or CF environments but is an essential feature of moving within the DF. Most subjects tend to avoid making curved movements in the unstable DF [12], keeping their trajectory close to the straight line [10]. It is clear that somatosensory information is sufficient to allow the subjects to perform in this manner.

Previous studies have shown that visual feedback of hand position throughout the movement is used to correct the hand trajectory at relatively short delays [37]–[40]. Visual feedback of the hand trajectory has been shown to reduce the endpoint variability of reaching movements [41]–[43]. However these studies have not examined the variability throughout the movement. When this was examined with data in the NF and CF fields, we found that with visual feedback of the hand position during the movements, the variability towards the end of the movement (approximately 70% of the movement distance or 75% percent of the movement time) decreases. Prior to this point, the variability of the movements both with and without visual feedback were similar. This suggests that the action of the visual feedback during movements normally occurs towards the end of movements. It is perhaps this visual feedback which gives rise to some of the corrective sub-movements which occur towards the end of slow or accurate movements [44]–[46] and the reduction in the variability of movements in the last quarter of movements to small targets [47]. A related possibility is that the presence of visual information improves the state estimate of the hand position [48]. It has previously been shown that visual feedback of the target provides a signal for correction of the hand trajectory even under conditions where the hand is not visible and the target shift is not perceived [43], [49], [50]. This would suggest that an estimate of the current state of the limb/hand position is maintained through a combination of the delayed available sensory feedback and efferent copy. Errors between the visual target and the estimated state of the limb produce corrective responses to ensure the hand reaches the target. In the case of visual feedback of the hand's location, the limb estimate is improved, resulting in reduced endpoint variability towards the end of the movements.

### Proprioception and Vision as the Learning Signal

In goal-directed movements under normal circumstances visual and proprioceptive feedback can be integrated by the CNS in order to localize the arm and the target and to track the execution of movement. It has been found that this multi-modal sensory integration is done so that each mode is optimally weighted and integrated in such a way as to minimize the effects of sensory noise in terms of direction-dependent finite precision and accuracy [1]–[4], [51], [52]. For example, proprioception has been found to be more precise in depth than in azimuth and vice versa for vision, thus depending on the position of the hand, proprioception and vision can have different weights [3]. However it has not been clear which of these signals are responsible for driving the learning of novel dynamics. Our work has shown that visual feedback is not required for learning of either stable or unstable dynamics. This suggests that it may be proprioception alone which is primarily responsible for the learning of the dynamics. Previous research has shown that visual information appears to be responsible for learning the direction of the movement and path planning [19], supporting the idea that the direction of movement and the joint torques/muscle forces are planned separately in the brain [6], [53]. This is further supported by the studies on humans without either vision or proprioception. Subjects without proprioception are able to adapt to visuomotor rotations [54] suggesting that the visual signal is enough for the re-mapping of movement direction planning. However, when we examine adaptation to novel dynamics a different story emerges. The absence of visual feedback still allows congenitally blind subjects to make straight movements and adapt to novel dynamic force fields [21]. On the other hand, subjects without proprioception are unable to learn the correct muscle activation patterns to adapt to their self produced joint interaction torques during reaching [55]–[57] although these effects could be due to an inability to use proprioception in order to appropriately time initiation of the sequences of the out and back movements [58]. Visual feedback does provide useful information for dynamical control, in particular to select different internal models of objects and to provide some useful information to update the internal model during reaching [56], [59]. However, while visual feedback may predominately affect the learning and re-mapping of path planning, it appears that proprioceptive feedback predominately drives the learning and generalization of dynamics.

The sensorimotor system's dependence on somatosensory rather than visual information for the learning of dynamics may be unsurprising when considering the transformations required to determine the appropriate change in the feedforward motor command based on an error signal from each of these sensory systems[60]. An error detected in the visual system requires extensive information on the posture of the limb in order to determine the appropriate muscles to activate to compensate for the disturbance produced by the novel dynamics. In contrast, the stretch reflexes induced in the stretched or shortened muscles already contain information on which muscles are required to compensate for the disturbance. Long latency stretch reflexes already produce a coordinated response to the perturbation [61]–[63]. We are currently working on a simple dynamics adaptation algorithm based upon the stretch of each muscle during adaptation which may also explain the predominate dependence on proprioceptive feedback for the adaptation to both stable and unstable dynamics.
